# Numerical Study on Generalized Heat and Mass in Casson Fluid with Hybrid Nanostructures

**DOI:** 10.3390/nano11102675

**Published:** 2021-10-11

**Authors:** Muhammad A. Sadiq, Haitham M. S. Bahaidarah

**Affiliations:** 1 Department of Mathematics, DCC-KFUPM, P.O. Box 5084, Dhahran 31261, Saudi Arabia; 2 Interdisciplinary Research Center for Hydrogen and Energy Storage, KFUPM, Dhahran 31261, Saudi Arabia; 3 Mechanical Engineering Department, King Fahd University of Petroleum and Minerals, Dhahran 31261, Saudi Arabia; haithamb@kfupm.edu.sa

**Keywords:** yield stress, thermal relaxation time, generalized Fick’s law, hybrid nanostructures, thermal conductivity effectiveness

## Abstract

The rheological model for yield stress exhibiting fluid and the basic laws for fluid flow and transport of heat and mass are used for the formulation of problems associated with the enhancement of heat and mass due to dispersion of nanoparticles in Casson. The heat and mass transfer obey non-Fourier’s laws and the generalized Fick’s law, respectively. Model problems are incorporated by thermal relaxation times for heat and mass. Transfer of heat energy and relaxation time are inversely proportional, and the same is the case for mass transport and concentration relaxation time. A porous medium force is responsible for controlling the momentum thickness. The yield stress parameter and diffusion of momentum in Casson fluid are noticed to be inversely proportional with each other. The concentration gradient enhances the energy transfer, and temperature gradient causes an enhancement diffusion of solute in Casson fluid. FEM provides convergent solutions. The relaxation time phenomenon is responsible for the restoration of thermal and solutal changes. Due to that, the thermal and solutal equilibrium states can be restored. The phenomenon of yield stress is responsible for controlling the momentum boundary layer thickness. A porous medium exerts a retarding force on the flow, and therefore, a deceleration in flow is observed. The thermal efficiency of $MoS_{2}-SiO_{2}-$ *Casson fluid* is greater than the thermal efficiency of $SiO_{2}-$ *Casson fluid*.

## 1. Introduction

The diverse rheological behavior of non-Newtonian fluid motivated researchers to propose diverse rheological models. Each model captures some specific rheological features. Casson rheological model is a non-Newtonian rheological model and captures yield stress rheological features. This means, to some extent, Casson fluid behaves like a solid and, after some specific value of applied stress, it starts behaving like a fluid. This specific value of applied stress is called yield stress. The simplest rheological model for the incompressible flow of Casson fluid is given by

(1) $$\tau =-PI+\left(1+\frac{1}{\beta }\right)\mu A_{1}$$

where β is the Casson fluid parameter and for β→∞, Equation (1) reduces to the rheological tensor of an incompressible Newtonian fluid. Numerical studies on various aspects related to Casson fluid have been published so far. For example, Hamid et al. [1] wrote a study on the existence of dual solutions for the problem related to heat transfer in Casson fluid over the stretchable surface. Nadeem et al. [2] discussed the 3D flow of Casson fluid induced by wall moving with the exponential surface. Mukhopadhyay et al. [3] theoretically studied the transfer of heat energy in Casson fluid over a surface moving with nonlinear velocity. Hayat et al. [4] examined the effects of temperature gradient and concentration gradients on mass and heat transfer in Casson fluid, respectively. Khan et al. [5] examined the simultaneous effects of homogeneous–heterogeneous chemical reactions on mass transfer in Casson fluid. The author has carefully reviewed the literature and has come to know that no study using hybrid nanostructures ($MoS_{2}$ and $SiO_{2}$) in Casson fluid with the novel model (given by Equations (2)–(5)) is published yet.

The energy crisis is the biggest challenge to humankind. Energy storage and its efficient utilization is the need of the hour. Techniques for minimizing energy losses are highly required. With this need in mind, researchers working in the field of thermal and cooling systems have been striving for introducing novel techniques for efficient transport of heat energy and the efficient usage of stored energy. Recently, a technique of inclusion of nanosized solid structures in the fluid has been introduced to obtain the fluid mixtures of very good thermal conductivity. Such mixtures serve as better working fluids, and consequently, the thermal performance of the fluid increases. Because of numerous applications of fluids with nanoparticles, researchers have studied much of the dynamics of such fluids. For instance, Sheikholeslami et al. [6] investigated the effects of nanoparticles $Fe_{3}O_{4}\;$ on heat transfer in water. Sheikholeslami et al. [7] conducted finite element numerical experiments for the analysis of thermal enhancement in fluid subjected to the variable magnetic field. Dogonchi et al. [8] also analyzed the impact of nanoparticles and thermal radiations on the transfer of heat in the mixed convection flow of fluid over a moving body. Dogonchi et al. [9] studied the role of Cu− nanoparticles on the enhancement of heat energy in the presence of homogeneous–heterogeneous chemical reactions in water subjected to the uniform heat source. Ayub et al. [10] used novel heat flux models for the modeling of heat transfer in viscoelastic fluid with nanoparticles. Rana et al. [11] used Koo-Kleinstreuer and Li models with novel heat flux theory to model heat transfer in Sutter by fluid with nanoparticles. Studies [6,7,8,9,10,11] and the references therein are limited to thermal enhancement via mono-nanoparticles. The recent advancement in the field reveals that the better method is the inclusion of nanoparticles of more than one kind. Therefore, several studies have been published. The reasoning and justification are given below.

The advancement in technologies and success in the synthesis of nanosized particles has brought a great revolution for introducing a class of special class of fluids called hybrid nanofluids. These are fluids that are homogeneous mixtures of nanoparticles of more than one kind. It is a proven fact that hybrid nanofluids are better coolants in comparison to nanofluids. Due to this factual reason, hybrid nanofluids have been frequently studied. The recent advancement in the field of thermal enhancement has revealed that the dispersion of nanoparticles of more than one kind is more effective than the dispersion of nanoparticles of a single kind. The fluid with more than one nanoparticle is a hybrid nanofluid. Several studies on this topic have been published. For example, Ahmed et al. [12] studied the impact of hybrid nanoparticles on heat transfer in a pulsating flow exposed to the magnetic field and thermal radiations. Iftikhar et al. [13] discussed the role of Cu  and $SiO_{2}\;$ in enhancing heat transfer in water. Benkhedda et al. [14] studied the importance of hybrid nanoparticles in enhancing thermal transfer in fluid in a pipe. Aziz et al. [15] examined the role of hybrid nanoparticles on entropy generation in Erying–Powell fluid subjected linear thermal radiations and viscous dissipation. Subhani et al. [16] theoretically analyzed the impact of hybrid nanoparticles on heat-transfer enhancement in the micropolar fluid. Wiani et al. [17] discussed the role of hybrid nanoparticles on heat transfer in fluid moving on a curved surface. Kaneez et al. [18] numerically analyzed the influence of hybrid nanoparticles on thermal enhancement in Casson fluid with dust particles. It is important to mention that this study on an enhancement of heat in Casson fluid with hybrid nanoparticles. However, the present study is about the impact of hybrid nanoparticles on heat and mass transport in Casson fluid with hybrid nanoparticles. Further, the present work considers the flow and heat transfer in Casson fluid over a vertical surface. In this case, the buoyance force is significant and is considered here. This is another aspect due to which the present work differs from the work of Kaneez et. al. [18].

The electrically conducting fluids moving in the presence of magnetic fluid have been found in many natural and man-made applications. The flows of such fluids are called MHD flows and have been investigated a lot in the present era. For instance, Nawaz et al. [19] examined the impact of magnetic fields on the flow of micropolar fluid composed of ionized particles. Nawaz et al. [20] studied the magnetic field on the mass and heat diffusion in the Newtonian fluid between two walls moving with nonuniform velocity in the radial direction. Asif et al. [21] investigated the thermal performance of the fluid-exposed magnetic field. In light of these studies [19,20,21], the authors were inspired and considered how magnetic field applied to the flow because Casson fluid is an electrically conducting fluid and experiences Lorentz force arises due to the change in magnetic flux. Flows over vertical boundaries are greatly affected by a buoyancy force that arises due to density differences caused by temperature and concentration differences. Downward flows experience positive a buoyancy force, whereas upward flows experience a negative buoyancy force. Momentum equations are modified in terms of buoyancy force. Such flows are called mixed convective flows over moving boundaries. Such flows have been studied intensely in recent years. The suitable references are listed [22,23,24,25,26,27].

Brownian motion and thermophoretic effects affect flow characteristics and, therefore, are neglected. Several kinds of research have discussed these effects. For instance, Sheikholeslam et al. [28] studied the influence of thermophoresis and Brownian motion on heat and mass in a mixed convection flow of electrically conducting fluid containing copper nanoparticles and subjected to the magnetic field. Kandasamy et al. [29] also studied the influence of thermophoresis and Brownian motion on the simultaneous impact of heat and mass transfer subjected to the thermal stratification in fluid containing nanoparticles. Makinde et al. [30] examined the role of thermophoresis and Brownian motion on the biconvective flow of MHD fluid over a surface of paraboloid revolution. Lin et al. [31] discussed the influence of thermophoresis and Brownian motion on heat transfer in fluid in a groove rotating with constant angular velocity. Some further applications and related aspects are mentioned in recent published work. Non-newtonian casson fluid flow over a stretching, exponentially embedded surface was examined by Animasaun, I et al. [32] for the laminar free convective MHD boundary layer. The effect of all of the influencing factors on the velocity and temperature profiles of the flow at the boundary was studied with an approximate solution (HAM) approach. Alruwashid et al. [33] analyzed the effects of graphene concentration on the electrochemical properties of cobalt ferrite nanocomposite materials. Pal et al. [34] developed novel nanostructures using pentablock polymers. Nasr et al. [35] introduced the technique of surface enhancement for nanosheets. Zemtsova et al. [36] published a comprehensive study on the feature of the synthesis of the disposed TiC with a nickel nanostructures.

A comprehensive review of the literature has shown that no study on the impact of hybrid nanoparticles ($MOS_{2}-SiO_{2}$) on thermal performance of Casson under Brownian and thermophoretic effects has been conducted so far. Secondly, the researches on thermal enhancement are conducted numerically using numerical techniques other than FEM. FEM is a powerful method and is less constrained. It converges rapidly, and the accuracy of results is quite sound. The stated problems are solved numerically by FEM. Further, no author has discussed the Brownian motion and thermophoretic mechanism in the presence of hybrid nanofluid so far. This means the impact of dispersion of nanoparticles on simultaneous heat and mass transfer has not been studied yet. This research is arranged in five sections. Section 1 is related to the background. Section 2 contains the formulation of problems. Section 3 discusses numerical methodology. Section 4 is about the discussion of results based on numerical simulations. Section 5 contains the key results of this study.

## 2. Formulation of Problem

The boundary layer approximations give the following governing models:

(2) $$\frac{\partial u}{\partial x}+\frac{\partial v}{\partial y}=0$$

(3) $$u\frac{\partial u}{\partial x}+v\frac{\partial u}{\partial y}=\frac{1}{\rho_{hnf}}\left(\mu_{hnf}+\frac{p_{y}}{\sqrt{2\pi_{c}}}\right)\frac{\partial^{2}u}{\partial^{2}y}+{\left(\beta_{hnf}\right)}_{T}g\left(T-T_{\infty }\right)+{\left(\beta_{hnf}\right)}_{C}g\left(C-C_{\infty }\right)-\frac{\sigma_{hnf}B_{0}^{2}u}{\rho_{hnf}}-\mu_{hnf}\frac{u}{K_{1}}$$

(4) $$u\frac{\partial T}{\partial x}+v\frac{\partial T}{\partial y}+\tau_{0}[\begin{array}{c} u\frac{\partial^{2}T}{\partial x^{2}}+v\frac{\partial^{2}T}{\partial y^{2}}+u\frac{\partial v}{\partial x}\frac{\partial T}{\partial y}+v\frac{\partial u}{\partial y}\frac{\partial T}{\partial x}+2uv\frac{\partial^{2}T}{\partial x\partial y}-\frac{Q_{0}}{{\left(\rho c_{p}\right)}_{hnf}}\left(u\frac{\partial T}{\partial x}-v\frac{\partial T}{\partial y}\right)\\ -\frac{1}{{\left(\rho c_{p}\right)}_{hnf}}\left(\mu_{hnf}+\frac{p_{y}}{\sqrt{2\pi_{c}}}\right)\frac{\partial u}{\partial x}\left(u\frac{\partial u}{\partial x}-v\frac{\partial^{2}u}{\partial y^{2}}\right)-\frac{\sigma_{hnf}B_{0}^{2}}{{\left(\rho c_{p}\right)}_{hnf}}\left(u\frac{\partial^{2}u}{\partial y^{2}}-v\frac{\partial^{2}u}{\partial x\partial y}\right) \end{array}]\;=\frac{K_{hnf}}{{\left(\rho c_{p}\right)}_{hnf}}\frac{\partial^{2}u}{\partial^{2}y}+\frac{1}{{\left(\rho c_{p}\right)}_{hnf}}\left(\mu_{hnf}+\frac{p_{y}}{\sqrt{2\pi_{c}}}\right){\left(\frac{\partial u}{\partial y}\right)}^{2}+\frac{\sigma_{hnf}B_{0}^{2}u^{2}}{{\left(\rho c_{p}\right)}_{hnf}}+\frac{Q_{0}}{{\left(\rho c_{p}\right)}_{hnf}}\left(T-T_{\infty }\right)$$

(5) $$u\frac{\partial C}{\partial x}+v\frac{\partial C}{\partial y}+\tau_{1}\left(u\frac{\partial^{2}C}{\partial x^{2}}+v\frac{\partial^{2}C}{\partial y^{2}}+u\frac{\partial v}{\partial x}\frac{\partial C}{\partial y}+v\frac{\partial u}{\partial y}\frac{\partial C}{\partial x}+2uv\frac{\partial^{2}C}{\partial x\partial y}\right)=D_{hnf}\frac{\partial^{2}C}{\partial^{2}y}$$

**Figure 1:**
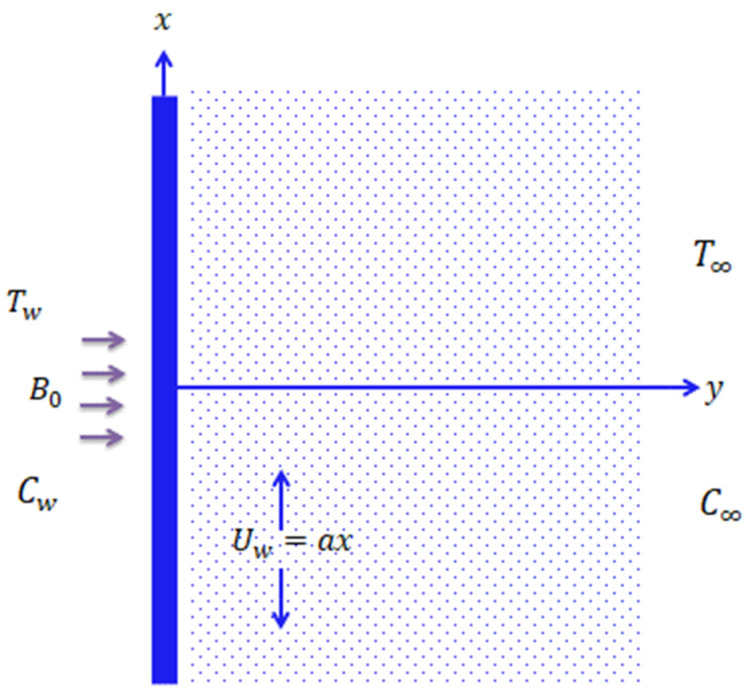
Flow configuration model.

The conditions implemented to solve the above system of PDEs are

(6) $$\begin{array}{c} u\left(x,0\right)=ax,v\left(x,0\right)=0,-\gamma_{0}k_{f}\frac{\partial T}{\partial y}\left(x,0\right)=h_{f}(T-T\left(x,0\right),T\left(x,\infty \right)=T_{\infty }\\ -\gamma_{0}D_{f}\frac{\partial C}{\partial y}\left(x,0\right)=h_{c}\left(C-C\left(x,0\right)\right),u\left(x,\infty \right)=0,C\left(x,\infty \right)=C_{\infty }, \end{array}\}$$

where [u,v,0] is the velocity; g is the gravitational acceleration; ρ  is the density; μ is the kinematic viscosity and $c_{p}\;$ specific heat constant; k  is the thermal conductivity; the subscript hnf stands for hybrid nanofluid; D is the mass diffusion coefficient; $\tau_{0}\;$ is the thermal relaxation time; $\tau_{1}\;$ is the concentration relaxation time; and $\gamma_{0}$ is the parameter associated with thermal slip. The flow diagram of the problem is represented in Figure 1. The following model for nanoparticles is incorporated

(7) $$\frac{\rho_{hnf}}{\rho_{f}}=\left(1-\varphi_{2}\right)\left\{\left(1-\varphi_{1}\right)+\frac{\varphi_{1}\rho_{s_{1}}}{\rho_{f}}\right\}+\frac{\varphi_{2}\rho_{s_{2}}}{\rho_{f}}$$

$$\frac{{\left(\rho c_{p}\right)}_{hnf}}{{\left(\rho c_{p}\right)}_{f}}=\left(1-\varphi_{2}\right)\left\{\left(1-\varphi_{1}\right)+\frac{\varphi_{1}{\left(\rho c_{p}\right)}_{s_{1}}}{{\left(\rho c_{p}\right)}_{f}}\right\}+\frac{\varphi_{2}{\left(\rho c_{p}\right)}_{s_{2}}}{{\left(\rho c_{p}\right)}_{f}}$$

$$\frac{\mu_{hnf}}{\mu_{f}}=\frac{1}{{\left(1-\varphi_{1}\right)}^{2.5}{\left(1-\varphi_{2}\right)}^{2.5}}$$

$$\frac{\sigma_{hnf}}{\sigma_{bf}}=\frac{\sigma_{s_{2}}+\left(n-1\right)\sigma_{bf}-\left(n-1\right)\varphi_{2}\left(\sigma_{bf}-\sigma_{s_{2}}\right)}{\sigma_{s_{2}}+2\sigma_{bf}+\varphi_{2}\left(\sigma_{bf}-\sigma_{s_{2}}\right)},\;\frac{\sigma_{bf}}{\sigma_{f}}=\frac{\sigma_{s_{1}}+2\sigma_{f}-\left(n-1\right)\varphi_{1}\left(\sigma_{f}-\sigma_{s_{1}}\right)}{\sigma_{s_{1}}+2\sigma_{f}+\varphi_{1}\left(\sigma_{f}-\sigma_{s_{1}}\right)}$$

$$\frac{k_{hnf}}{k_{bf}}=\frac{k_{s_{2}}+2k_{bf}-\left(n-1\right)\varphi_{2}\left(k_{bf}-k_{s_{2}}\right)}{k_{s_{2}}+2k_{bf}+\varphi_{2}\left(k_{bf}-k_{s_{2}}\right)},\;\frac{k_{bf}}{k}=\frac{k_{s_{1}}+2k_{f}-\left(n-1\right)\varphi_{1}\left(k_{f}-k_{s_{1}}\right)}{k_{s_{1}}+2k_{f}+\varphi_{1}\left(k_{f}-k_{s_{1}}\right)}$$

$$\frac{D_{hnf}}{D_{f}}=\frac{1}{\left(1-\varphi_{1}\right)\left(1-\varphi_{2}\right)}$$

Equations (2)–(7) in dimensionless form can be changed via the following change of variable:

(8) $$\begin{array}{c} u=axf^{\prime }\left(\eta \right),\;v=-\sqrt{a\upsilon_{f}}f\left(\eta \right),\eta =\sqrt{\frac{a}{\upsilon_{f}}}y,\theta \left(\eta \right)=\frac{T-T_{\infty }}{T_{w}-T_{\infty }}\\ \psi \left(\eta \right)=\sqrt{a\upsilon_{f}}xf\left(\eta \right),\;\phi \left(\eta \right)=\frac{C-C_{\infty }}{C_{w}-C_{\infty }} \end{array}\}$$

Hence, one obtains:

(9) $$\begin{array}{c} \frac{\upsilon_{hnf}}{\nu_{f}}\left(1+\frac{1}{\beta }\left(\frac{\mu_{f}}{\mu_{hnf}}\right)\right)f^{\prime \prime \prime }-[{\left(f^{\prime }\right)}^{2}-ff^{\prime \prime }]+{\left(Gr\right)}_{t}\theta \left(\eta \right)+{\left(Gr\right)}_{C}\phi \left(\eta \right)\\ -M\left(\frac{\sigma_{hnf}}{\sigma_{f}}\right)\left(\frac{\rho_{f}}{\rho_{hnf}}\right)f^{\prime }-\frac{\mu_{hnf}}{\mu_{f}}Kf^{\prime }=0 \end{array}\}$$

(10) $$\begin{array}{c} \left(\frac{k_{hnf}}{k_{f}}\right)\left(\frac{\rho_{f}}{\rho_{hnf}}\right)\left(\frac{{\left(C_{p}\right)}_{f}}{{\left(C_{p}\right)}_{hnf}}\right)\theta^{\prime \prime }+Prf\theta^{\prime }+\left(\frac{\rho_{f}}{\rho_{hnf}}\right)\left(\frac{{\left(C_{p}\right)}_{f}}{{\left(C_{p}\right)}_{hnf}}\right)Pr\beta^{*}\theta -\gamma Prf^{2}\theta^{\prime \prime }\\ -\gamma Prff^{\prime }\theta^{\prime }-\left(\frac{\rho_{f}}{\rho_{hnf}}\right)\left(\frac{{\left(C_{p}\right)}_{f}}{{\left(C_{p}\right)}_{hnf}}\right)\gamma Pr\beta^{*}f\theta^{\prime }+\left(\frac{\upsilon_{hnf}}{\nu_{f}}\right)\left(\frac{{\left(C_{p}\right)}_{f}}{{\left(C_{p}\right)}_{hnf}}\right)\left(1+\frac{1}{\beta }\left(\frac{\mu_{f}}{\mu_{hnf}}\right)\right)PrEc{\left(f^{\prime \prime }\right)}^{2}\\ +\left(\frac{\sigma_{hnf}}{\sigma_{f}}\right)\left(\frac{{\left(C_{p}\right)}_{f}}{{\left(C_{p}\right)}_{hnf}}\right)\left(\frac{\rho_{f}}{\rho_{hnf}}\right)MPrEc{\left(\;f^{\prime }\right)}^{2}\\ -2\left(\frac{\upsilon_{hnf}}{\nu_{f}}\right)\left(\frac{{\left(C_{p}\right)}_{f}}{{\left(C_{p}\right)}_{hnf}}\right)\left(1+\frac{1}{\beta }\left(\frac{\mu_{f}}{\mu_{hnf}}\right)\right)\gamma PrEc\left\{f\;f^{\prime \prime }f^{\prime \prime \prime }-f^{\prime }{\left(f^{\prime \prime }\right)}^{2}\right\}\\ -2\left(\frac{\upsilon_{hnf}}{\nu_{f}}\right)\left(\frac{{\left(C_{p}\right)}_{f}}{{\left(C_{p}\right)}_{hnf}}\right)\gamma MEcPr\left\{ff^{\prime }f^{\prime \prime }-{\left(f^{\prime }\right)}^{3}\right\}=0 \end{array}\}$$

(11) $$\frac{D_{hnf}}{D_{f}}\phi^{\prime \prime }+Scf\phi^{\prime }-\gamma_{1}Scf^{2}\phi^{\prime \prime }-\gamma_{1}Scff^{\prime }\phi^{\prime }=0$$

The associated boundary conditions are dimensionalized, and hence, one obtains:


(12) $$\begin{array}{c} f^{\prime }\left(0\right)=1,\;f\left(0\right)=0,\;\theta^{\prime }\left(0\right)=\frac{Bi}{\gamma_{0}}\left(1-\theta \left(0\right)\right),\;\phi^{\prime }\left(0\right)=\frac{Ei}{\gamma_{0}}\left(1-\phi \left(0\right)\right)\;\\ f^{\prime }\left(\infty \right)\to 0,\;\theta \left(\infty \right)\to 0,\;\phi \left(\infty \right)\to 0\; \end{array}\}$$

Derivatives concerning η  are denoted by prime. M is a magnetic parameter; ${\left(Gr\right)}_{t}\;and\;{\left(Gr\right)}_{c}\;$ are the Grashof numbers for temperature and concentration, respectively; $\beta^{*}$ is heat generation parameter; K is the porous medium parameter; Pr is the Prandtl-number; Sc is the Schmidt number; Ec is the Eckert number; γ  is the thermal relaxation time parameter; $\gamma_{1}\;$ is the concentration relaxation time parameter; and $B_{i}$ and $E_{i}$ are Biot numbers. These are expressed as

(13) $$\begin{array}{c} {\left(Gr\right)}_{t}=\frac{g\left(T_{w}-T_{\infty }\right){\left(\beta_{hnf}\right)}_{T}}{a^{2}x},{\left(Gr\right)}_{c}=\frac{g\left(C_{w}-C_{\infty }\right){\left(\beta_{hnf}\right)}_{C}}{a^{2}x},M=\frac{\sigma_{f}B_{0}^{2}}{\rho_{f}a},K=\frac{\mu_{f}}{aK_{1}}\\ Ec=\frac{a^{2}x^{2}}{c_{p}\left(T_{w}-T_{\infty }\right)},\;Pr=\frac{\mu_{f}{\left(c_{p}\right)}_{f}}{k_{f}},\beta^{*}=\frac{Q_{0}}{a{\left(\rho c_{p}\right)}_{f}},D_{f}=\frac{Dk_{T}\left(C_{w}-C_{\infty }\right)}{C_{s}C_{p}\left(T_{w}-T_{\infty }\right)},\\ Sc=\frac{\upsilon_{f}}{D_{f}}\;,Bi=\frac{h_{f}}{k_{f}}\sqrt{\frac{\upsilon_{f}}{a}},\gamma =\tau_{0}a,\gamma_{1}=\tau_{1}a,\;Ei=\frac{h_{c}}{D_{f}}\sqrt{\frac{\upsilon_{f}}{a}} \end{array}\}$$

The skin friction and Nusselt and Sherwood numbers are obtained as

(14) Cf=τxy|y=0ρfU02=f″(0)Rex12(1−φ1)2.5(1−φ2)2.5,Nu=−xkhnf∂T∂y|y=0kf(Tw−T∞)=−Rex12khnfkfθ′(0),Sh=−xDhnf∂C∂y|y=0Df(Cw−C∞)=−Rex12(1−φ1)(1−φ2)ϕ′(0),}

where $\tau_{w}$ is the wall shear stress and ${Re}_{x}=\frac{ax^{2}}{\nu_{f}}$ is the Reynolds number.

Thermophysical properties are tabulated in Table 1.

## 3. Numerical Procedure

The different numerical schemes can be applied for the solution of Equations (8)–(12). However, FEM is powerful and provides the most accurate numerical solutions. The main steps are mentioned below.

**Step 1: Residuals are multiplied by weight functions and are integrated over the typical element.**

Linear weight and shape functions are given by

(15) $$S_{j}={\left(-1\right)}^{j-1}\left(\frac{\xi_{j+1}-\xi }{\xi_{j+1}-\xi_{j}}\right),i=1,2$$

The dependent unknowns are approximated over the element $\left[\eta_{e},\eta_{e+1}\right]$ by the finite element approximations

$$f=\sum_{j=1}^{2}S_{j}f_{j},\theta =\sum_{j=1}^{2}S_{j}\theta_{j},\phi =\sum_{j=1}^{2}S_{j}\phi_{j},h=\sum_{j=1}^{2}S_{j}h_{j},$$

where $f_{j}$, $h_{j},\theta_{j}$ and $\phi_{j}$ are to be computed. $S_{j}$ is the shape function.

**Step 2: The weighted integrals are evaluated only for second-order linear terms to obtain weak forms of residuals.**

(16) $$\int_{\eta_{e}}^{\eta_{e+1}}w_{i}\left(f^{\prime }-h\right)d\eta =0,$$

(17) $$\int_{\eta_{e}}^{\eta_{e+1}}w_{i}\left(\begin{array}{c} \frac{\upsilon_{hnf}}{\nu_{f}}\left(1+\frac{1}{\beta }\left(\frac{\mu_{f}}{\mu_{hnf}}\right)\right)h^{\prime \prime }-[{\left(h\right)}^{2}-fh^{\prime }]+{\left(Gr\right)}_{T}\theta \left(\eta \right)\\ +{\left(Gr\right)}_{C}\phi \left(\eta \right)-M\left(\frac{\rho_{f}}{\rho_{hnf}}\right)\left(\frac{{\left(C_{p}\right)}_{f}}{{\left(C_{p}\right)}_{hnf}}\right)h-\frac{\mu_{hnf}}{\mu_{f}}Kh \end{array}\right)d\eta =0\}$$

(18) $$\int_{\eta_{e}}^{\eta_{e+1}}w_{i}(\begin{array}{c} \left(\frac{k_{hnf}}{k_{f}}\right)\left(\frac{\rho_{f}}{\rho_{hnf}}\right)\left(\frac{{\left(C_{p}\right)}_{f}}{{\left(C_{p}\right)}_{hnf}}\right)\theta^{\prime \prime }+\left(\frac{\rho_{f}}{\rho_{hnf}}\right)\left(\frac{{\left(C_{p}\right)}_{f}}{{\left(C_{p}\right)}_{hnf}}\right)Pr\beta^{*}\theta -\gamma Prf^{2}\theta^{\prime \prime }\\ -\gamma Prfh\theta^{\prime }-\left(\frac{\rho_{f}}{\rho_{hnf}}\right)\left(\frac{{\left(C_{p}\right)}_{f}}{{\left(C_{p}\right)}_{hnf}}\right)\gamma Pr\beta^{*}\theta^{\prime }+Prf\theta^{\prime }\\ +\left(\frac{\upsilon_{hnf}}{\nu_{f}}\right)\left(\frac{{\left(C_{p}\right)}_{f}}{{\left(C_{p}\right)}_{hnf}}\right)\left(1+\frac{1}{\beta }\left(\frac{\mu_{f}}{\mu_{hnf}}\right)\right)PrEc{\left(h^{\prime }\right)}^{2}\\ +\left(\frac{\sigma_{hnf}}{\sigma_{f}}\right)\left(\frac{{\left(C_{p}\right)}_{f}}{{\left(C_{p}\right)}_{hnf}}\right)\left(\frac{\rho_{f}}{\rho_{hnf}}\right)MPrEc{\left(h\right)}^{2}\\ -2\left(\frac{\upsilon_{hnf}}{\nu_{f}}\right)\left(\frac{{\left(C_{p}\right)}_{f}}{{\left(C_{p}\right)}_{hnf}}\right)\left(1+\frac{1}{\beta }\left(\frac{\mu_{f}}{\mu_{hnf}}\right)\right)\gamma PrEcf\;h^{\prime }h^{\prime \prime }\\ -2\left(\frac{\upsilon_{hnf}}{\nu_{f}}\right)\left(\frac{{\left(C_{p}\right)}_{f}}{{\left(C_{p}\right)}_{hnf}}\right)\gamma MEcPr\left\{fhh^{\prime }-{\left(h\right)}^{3}\right\}\\ +2\left(\frac{\upsilon_{hnf}}{\nu_{f}}\right)\left(\frac{{\left(C_{p}\right)}_{f}}{{\left(C_{p}\right)}_{hnf}}\right)\left(1+\frac{1}{\beta }\left(\frac{\mu_{f}}{\mu_{hnf}}\right)\right)\gamma PrEch{\left(h^{\prime }\right)}^{2} \end{array})d\eta =0\}$$

(19) $$\int_{\eta_{e}}^{\eta_{e+1}}w_{i}\left(\frac{D_{hnf}}{D_{f}}\phi^{\prime \prime }+Scf\phi^{\prime }-\gamma_{1}Scf^{2}\phi^{\prime \prime }-\gamma_{1}Scfh\phi^{\prime }\right)d\eta =0$$

**Step 3: The weak forms are approximated via Galerkin approximations.**

$$K_{ij}^{11}=\int_{\eta_{e}}^{\eta_{e+1}}S_{i}S_{j}^{\prime }d\eta ,K_{ij}^{12}=\int_{\eta_{e}}^{\eta_{e+1}}-S_{i}S_{j}d\eta ,$$

$$K_{ij}^{22}=\int_{\eta_{e}}^{\eta_{e+1}}\left(\begin{array}{c} -\left(\frac{\upsilon_{hnf}}{\upsilon_{f}}\right)\left(1+\frac{1}{\beta }\left(\frac{\mu_{f}}{\mu_{hnf}}\right)\right)S_{i}^{\prime }S_{j}^{\prime }-\bar{h}S_{i}S_{j}+\bar{f}{\left(\bar{h}\right)}^{\prime }S_{i}S_{j}^{\prime }\\ -M\left(\frac{\sigma_{hnf}}{\sigma_{f}}\right)\left(\frac{\rho_{f}}{\rho_{hnf}}\right)S_{i}S_{j}-\frac{\mu_{hnf}}{\mu_{f}}KS_{i}S_{j} \end{array}\right)d\eta ,$$

$$K_{ij}^{23}=\int_{\eta_{e}}^{\eta_{e+1}}{\left(Gr\right)}_{t}S_{i}S_{j}d\eta ,K_{ij}^{24}=\int_{\eta_{e}}^{\eta_{e+1}}{\left(Gr\right)}_{c}S_{i}S_{j}d\eta ,$$

$$K_{ij}^{32}=\int_{\eta_{e}}^{\eta_{e+1}}(\begin{array}{c} \left(\frac{\upsilon_{hnf}}{\nu_{f}}\right)\left(\frac{{\left(C_{p}\right)}_{f}}{{\left(C_{p}\right)}_{hnf}}\right)\left(1+\frac{1}{\beta }\left(\frac{\mu_{f}}{\mu_{hnf}}\right)\right)PrEc{\left(\bar{h}\right)}^{\prime }S_{i}S_{j}^{\prime }+MEc\left(\frac{\sigma_{hnf}}{\sigma_{f}}\right)\left(\frac{{\left(C_{p}\right)}_{f}}{{\left(C_{p}\right)}_{hnf}}\right)\\ +\left(\frac{\rho_{f}}{\rho_{hnf}}\right)PrS_{i}S_{j}-2\left(\frac{\upsilon_{hnf}}{\nu_{f}}\right)\left(\frac{{\left(C_{p}\right)}_{f}}{{\left(C_{p}\right)}_{hnf}}\right)\left(1+\frac{1}{\beta }\left(\frac{\mu_{f}}{\mu_{hnf}}\right)\right)\gamma PrEc{\left(\bar{h}\right)}^{\prime }S_{i}^{\prime }S_{j}^{\prime }\\ +2\left(\frac{\upsilon_{hnf}}{\nu_{f}}\right)\left(\frac{{\left(C_{p}\right)}_{f}}{{\left(C_{p}\right)}_{hnf}}\right)\left(1+\frac{1}{\beta }\left(\frac{\mu_{f}}{\mu_{hnf}}\right)\right)\gamma PrEc\bar{h}{\left(\bar{h}\right)}^{\prime }S_{i}S_{j}^{\prime }\\ -2\left(\frac{\upsilon_{hnf}}{\nu_{f}}\right)\left(\frac{{\left(C_{p}\right)}_{f}}{{\left(C_{p}\right)}_{hnf}}\right)\gamma MEcPr\bar{f}\bar{h}S_{i}S_{j}^{\prime }\\ +2\left(\frac{\upsilon_{hnf}}{\nu_{f}}\right)\left(\frac{{\left(C_{p}\right)}_{f}}{{\left(C_{p}\right)}_{hnf}}\right)\gamma MEcPr{\left(\bar{h}\right)}^{2}S_{i}S_{j} \end{array})d\eta ,\}$$

$$K_{ij}^{33}=\int_{\eta_{e}}^{\eta_{e+1}}(\begin{array}{c} -\left(\frac{K_{hnf}}{K_{f}}\right)\left(\frac{\rho_{f}}{\rho_{hnf}}\right)\left(\frac{{\left(C_{p}\right)}_{f}}{{\left(C_{p}\right)}_{hnf}}\right)S_{i}^{\prime }S_{j}^{\prime }+Pr\bar{f}S_{i}S_{j}^{\prime }+\left(\frac{\rho_{f}}{\rho_{hnf}}\right)\left(\frac{{\left(C_{p}\right)}_{f}}{{\left(C_{p}\right)}_{hnf}}\right)Pr\beta^{*}S_{i}S_{j}\\ +\gamma Pr{\left(\bar{f}\right)}^{2}S_{i}^{\prime }S_{j}^{\prime }-\gamma Pr\bar{f}\bar{h}S_{i}S_{j}^{\prime }-\left(\frac{\rho_{f}}{\rho_{hnf}}\right)\left(\frac{{\left(C_{p}\right)}_{f}}{{\left(C_{p}\right)}_{hnf}}\right)\gamma Pr\beta^{*}S_{i}S_{j}^{\prime } \end{array})d\eta ,\}$$

$$K_{ij}^{44}=\int_{\eta_{e}}^{\eta_{e+1}}\left(-\frac{D_{hnf}}{D_{f}}S_{i}^{\prime }S_{j}^{\prime }+Sc\bar{f}S_{i}S_{j}^{\prime }-\gamma_{1}Sc{\left(\bar{f}\right)}^{2}S_{i}^{\prime }S_{j}^{\prime }-\gamma_{1}Sc\bar{f}\bar{h}S_{i}S_{j}^{\prime }\right)d\eta ,$$

where $\bar{f}$ and $\bar{h}\;$ are nodal values that are computed.

**Step 4: Stiffness elements are derived, and the stiffness matrix is constructed under the assembly procedure**.

(20) [K{π}]{π}={F}

**Step 5: The nonlinear system is linearized.**

The nonlinear system is linearized by Picard linearization proposed as

(21) $$\left[K{\left\{\pi \right\}}^{r-1}\right]{\left\{\pi \right\}}^{r}=\left\{F\right\}$$

where ${\left\{\pi \right\}}^{r-1}$ is the nodal value at (r−1)th the iteration and ${\left\{\pi \right\}}^{r}$ is the nodal value at the rth iteration.

**Step 6: A grid-independent study is performed**.

The numerical solutions are also checked to be grid independent. This mesh-free analysis is recorded in Table 2. The recorded values in Table 2 show that the numerical results become grid independent when the computational domain [0, 12] is broken into two elemental line segments.

**Step-7: Results are validated**

The present modeled problems can be reduced to the case of already published work by Aninasaun et al. [32] when $\varphi_{1}=\varphi_{2}=0,\;{\left(Gr\right)}_{c}={\left(Gr\right)}_{t}=K=\gamma =\gamma_{1}=0$. The results are computed for this special case and are compared by the results obtained by Aninasaun et al. [32]. The comparative analysis is provided in Table 3.

## 4. Results and Discussion

The yield stress rheological model, non-Fourier law of heat conduction, and generalized Fick’s law are simultaneously used in the formulation of problems dealing with the simultaneous transfer of heat energy and mass in Casson fluid with hybrid nanoparticles over a vertical surface. The buoyancy forces due to the density differences under Boussinesq approximations are considered of a significant order of magnitude, and Lorentz force is measured from Ohm’s law when the magnetic Reynolds number is small. The numerical experiments with three samples of parametric values are performed. The simulations are visualized, and the outcomes are displayed in the form of graphs and numerical data. The mesh-free analysis is performed, and the results are validated. A detailed discussion about visualized outcomes is provided below.

**Figure 2:**
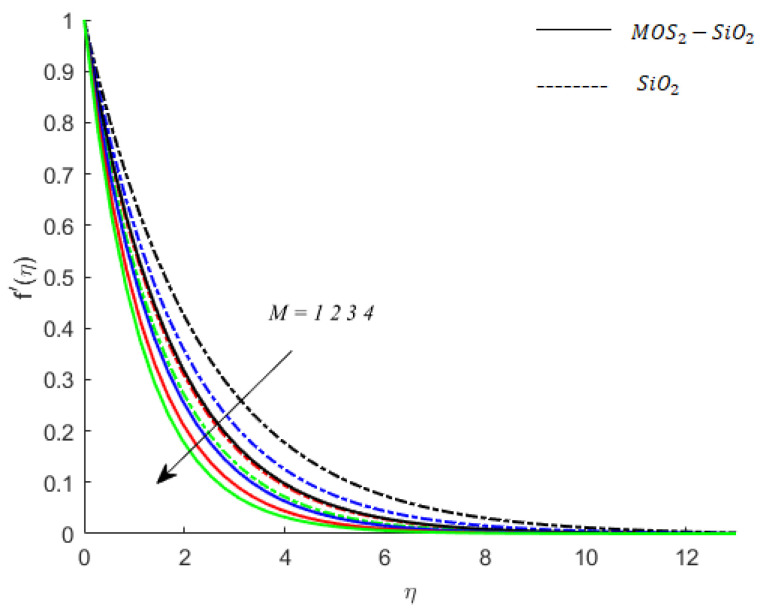
Influence of M on velocity when $Pr=0.9,Ec=0.2,Sc=0.8,K=0.1,Bi=0.05,\beta^{*}=0.1,\gamma =0.1,\gamma_{1}=0.1$.

**Fluid particles motion and magnetic field:** The Lorentz force is incorporated in the momentum equation through Ohm’s law and the term involved Hartmann number in the dimensionless equation (Equation (8)). This dimensionless number (M) is capable of determining the impact of the change of intensity of magnetic field on the motion of fluid particles. Since the Lorentz force is negative (in this case), an increase in M through values 1, 2, 3,4 depicts the retardation to the motion of fluid particles. Moreover, the impact of change of magnetic intensity on the motion of particles of both fluids ($MoS_{2}-SiO_{2}-Casson\mathrm{and}\;SiO_{2}-Casson\;fluid$) is examined, and the related outcomes are provided by Figure 2. The continuous curves are velocity curves associated with the motion of $MoS_{2}-SiO_{2}-Casson\;fluid$, whereas the dotted curves are related to the velocity curves of $\;SiO_{2}-Casson\;fluid$. For both types of fluids, the fluids particles are observed to experience retardation. However, the retardation experienced by $MoS_{2}-SiO_{2}-\;Casson\;fluid$ (hybrid nanofluid) is greater than that experienced by $SiO_{2}-Casson\;fluid$ (nanofluid). Hence, it can be concluded that the viscous region that $MoS_{2}-SiO_{2}-\;Casson\;fluid$ has is wider than the viscous region associated with the flow of $SiO_{2}-Casson\;fluid$ (see Figure 2).

**Figure 3:**
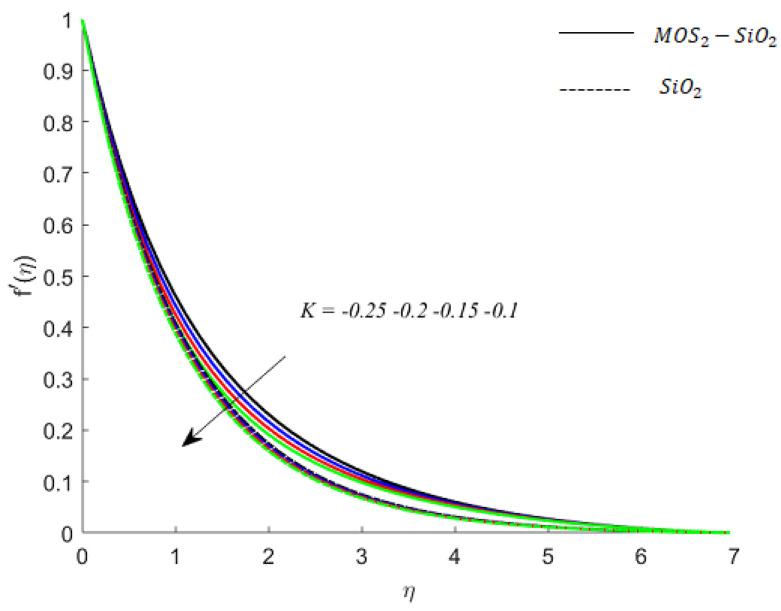
Influence of K on velocity when $M=0.9,Pr=0.9,Ec=0.2,Sc=0.8,Bi=0.05,\beta^{*}=0.1,\gamma =0.1,\gamma_{1}=0.1$.

**Fluid particles motion and resistance by the porous medium:** The parameter *K* is called the porous medium parameter, and its variation through increasing values (K=0.05, 0.1, 0.15, 0.2) depicts the decrease in voids between the particles of the porous medium. Therefore, the fluid particles of $MoS_{2}-SiO_{2}-\;Casson\;fluid$ and $SiO_{2}-Casson\;fluid$ face more a resistive force by the medium, and as a consequence, the motion of the particles of the fluid slows down. This fact is also visualized in numerical simulations (see Figure 3). It is also observed that $MoS_{2}-SiO_{2}-\;Casson\;fluid$ experiences more resistive force than $SiO_{2}-Casson\;fluid$. Numerical experiments have also predicted that the presence of a porous medium in the fluid regime may play a vital role in controlling the boundary layer thickness.

**Figure 4:**
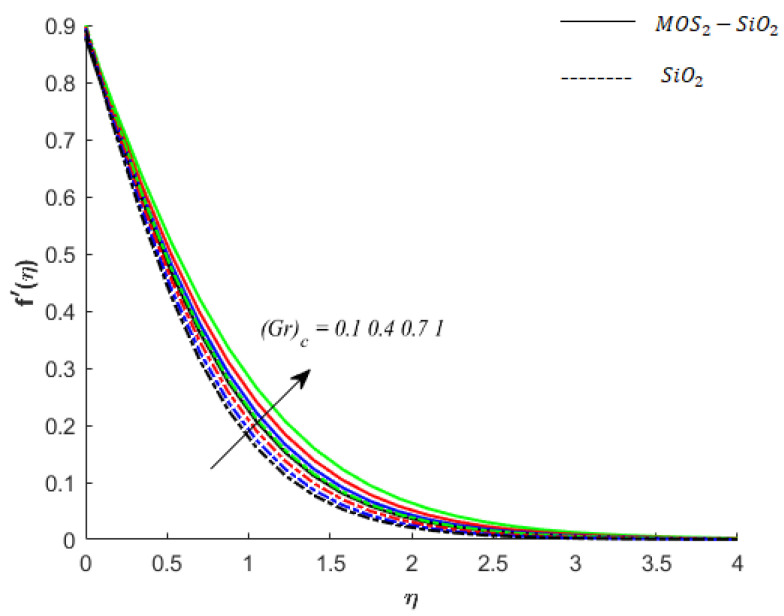
Influence of ${\left(Gr\right)}_{c}$ on velocity when $M=0.9,Pr=0.9,Ec=0.2,Sc=0.8,K=0.1,Bi=0.05,\beta^{*}=0.1,\gamma =0.1,\gamma_{1}=0.1$.

**Figure 5:**
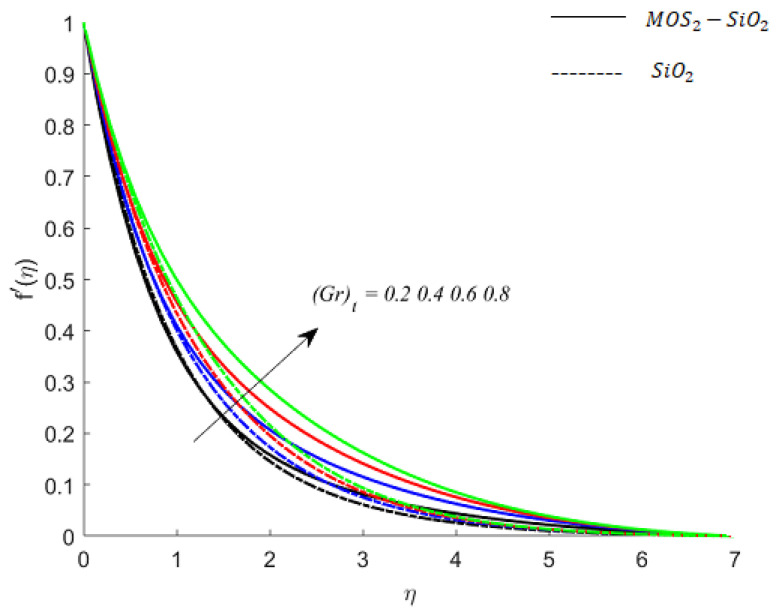
Influence of ${\left(Gr\right)}_{t}$ on velocity when $M=0.9,Pr=0.9,Ec=0.2,Sc=0.8,K=0.1,Bi=0.05,\beta^{*}=0.1,\gamma =0.1,\gamma_{1}=0.1$.

**Fluid particle motion and buoyancy force:** The parameters ${\left(Gr\right)}_{c}$ and ${\left(Gr\right)}_{t}$ are the coefficients of terms associated with buoyancy forces that arise due to the density differences caused by the compositional and temperature differences under Boussinesq approximation. For ${\left(Gr\right)}_{c}>0$ and ${\left(Gr\right)}_{t}$ >0, the buoyancy force is posture and assists the flow (see Figure 4 and Figure 5), whereas when ${\left(Gr\right)}_{c}$ <0  and ${\left(Gr\right)}_{t}$ <0, the Buoyancy force is negative and opposes the flow. This opposing behavior can be noticed during numerical experiments. However, the observations are not included here (due to the restrictions on the length of the article). It is also found that the buoyancy force in $MoS_{2}-SiO_{2}-Casson\;fluid$ is stronger than the buoyancy forces in $SiO_{2}-Casson\;fluid$.

**Figure 6:**
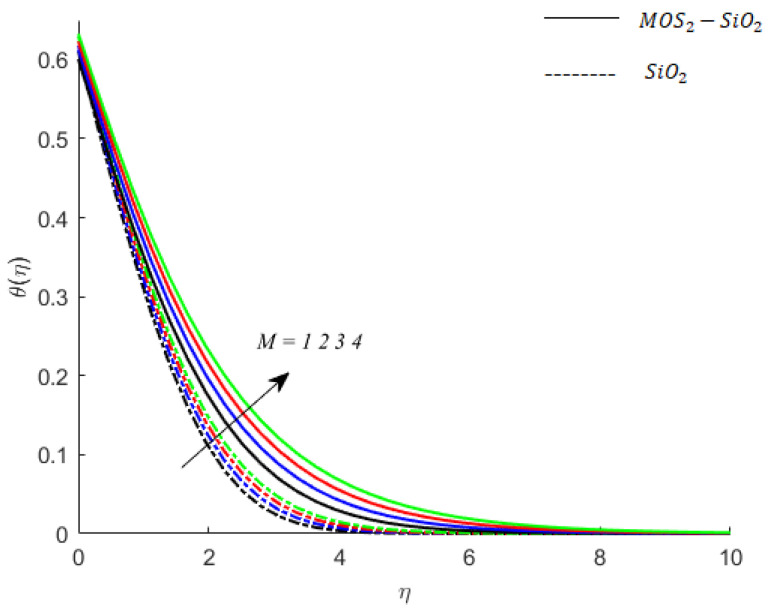
Influence of M on temperature when $Pr=0.9,Ec=0.2,Sc=0.8,K=0.1,Ei=0.1,Bi=0.05,\beta^{*}=0.1,\gamma =0.1,\gamma_{1}=0.1$.

**Temperature of fluid particles and intensity of magnetic field:** The $MoS_{2}-SiO_{2}-Casson$ and $\;SiO_{2}-Casson$ fluids are electrically conducting fluids. When electric current passes through $MoS_{2}-SiO_{2}-Casson$ and $SiO_{2}-Casson$ fluids, some of the electrical energy converts into heat, and this phenomenon is called Ohmic dissipation. This phenomenon is also considered here in this study. The term in dimensionless energy equation involving *M* is due to Joule heating (Ohmic heating) and determines the impact of Ohmic dissipation on the temperature of the fluid. This dissipated heat raises the kinetic energy of the fluid particles, and therefore, their temperature increases. This effect can be noticed in Figure 6. This figure also demonstrates that the Ohmic dissipation phenomenon in $MoS_{2}-SiO_{2}-Casson\;fluid$ is stronger than that in $\;SiO_{2}-Casson$. Therefore, the rise in temperature of fluid particles of $MoS_{2}-SiO_{2}-\mathrm{Casson}$ is greater than the rise of temperature of fluid particles of $SiO_{2}-Casson\;fluid$. Therefore, the influence of Ohmic dissipation on boundary layer thickness associated with the flow of $MoS_{2}-SiO_{2}-Casson\;fluid$ is greater than the influence of Ohmic dissipation on boundary layer thickness associated with the flow of $SiO_{2}-Casson\;fluid$.

**Figure 7:**
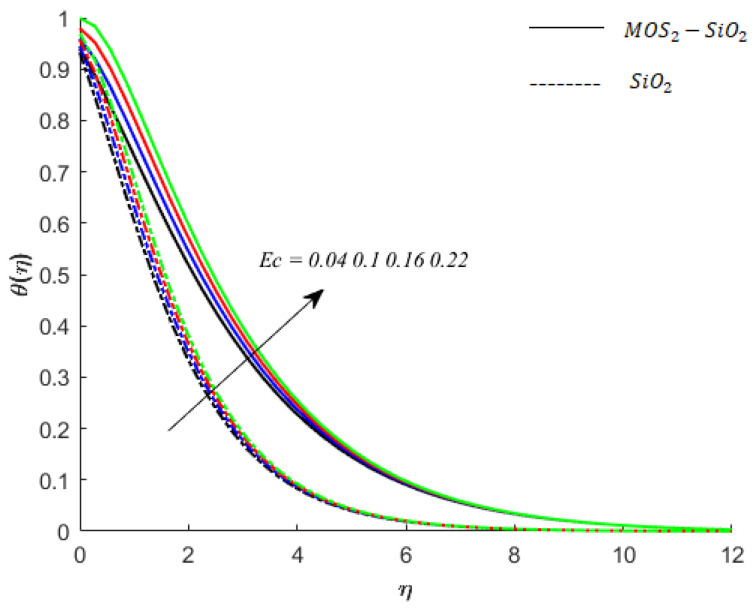
Influence of Ec on temperature when $M=0.9,Pr=0.9,Sc=0.8,K=0.1,Ei=0.1,Bi=0.05,\beta^{*}=0.1,\gamma =0.1,\gamma_{1}=0.1$.

**Temperature of fluid particles and viscous dissipation:** The friction among the particles of fluid causes the dissipation of heat which is equal to the rate at which the work is performed by friction force. This effect is called viscous dissipation. It is noticed during simulations that the inclusion of hybrid nanostructures ($MoS_{2}\;\mathrm{and}\;SiO_{2}$) increases the friction among the particles of the fluid. It can also be observed that the rise in friction among the fluid particles due to the inclusion of hybrid nanoparticles ($MoS_{2}-SiO_{2}$) is greater than the rise in friction among the particles of fluid due to the inclusion of $SiO_{2}$ in fluid (see Figure 7).

**Figure 8:**
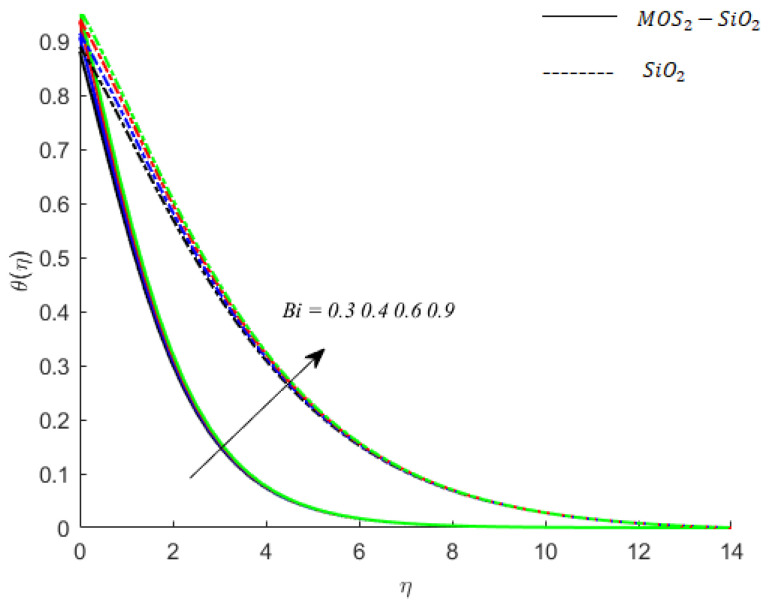
Influence of Bi on temperature when $M=0.9,Pr=0.9,Ec=0.2,Sc=0.8,Ei=0.1,K=0.1,\beta^{*}=0.1,\gamma =0.1,\gamma_{1}=0.1$.

**Temperature of fluid particles and Biot number:** The Biot number $B_{i}$ appears in the dimensionless form of convective boundary conditions. The role of $B_{i}$ on the temperature of fluid particles of $MoS_{2}-SiO_{2}-Casson\;fluid$ is examined. The comparative analysis between the role of $B_{i}$ on temperatures of $SiO_{2}-Casson\;fluid$ given by Figure 8. In both types of fluids (mono-nanofluid and hybrid nanofluid), temperature fluid particles have an increasing behavior for Biot number $B_{i}.$

**Figure 9:**
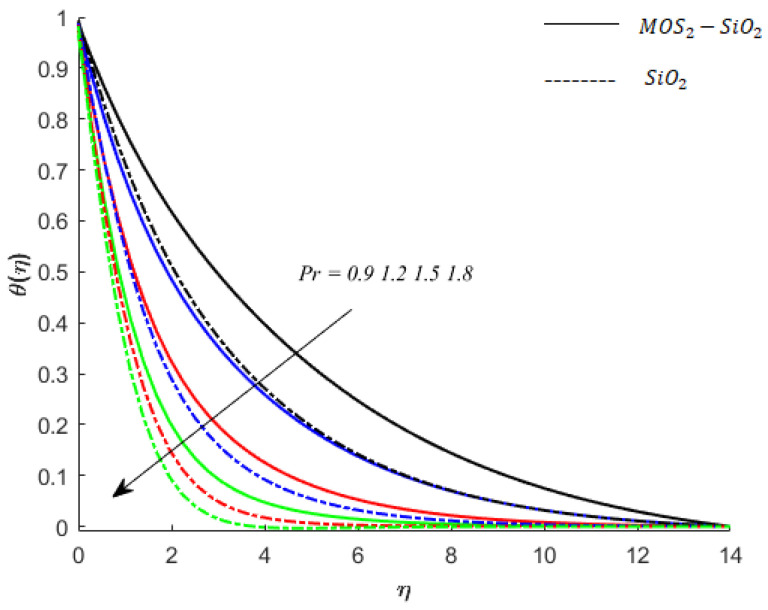
Influence of Pr on temperature when $M=0.9,Ec=0.2,Sc=0.8,K=0.1,Ei=0.1,Bi=0.05,\beta^{*}=0.1,\gamma =0.1,\gamma_{1}=0.1$.

**Temperature of fluid particles and Prandtl number:** The Pr is the dimensionless parameter, and an increase in the Pr relates to a decrease in thermal conductivity corresponding to the lower ability of fluid to conduct heat. Consequently, the temperature decreases. This decrease in temperature of $MoS_{2}-SiO_{2}-Casson\;fluid$ and $SiO_{2}-Casson\;fluid$ can be noticed (see Figure 9). The decreasing impact of Pr on the temperature of $SiO_{2}-Casson\;fluid$ is greater than the impact on the temperature of $MoS_{2}-SiO_{2}-Casson\;fluid.$

**Figure 10:**
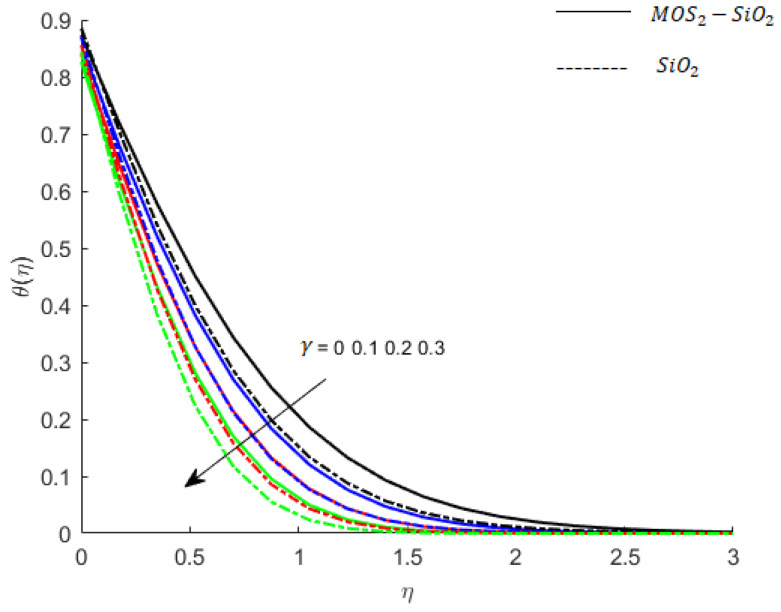
Influence of γ on temperature when $M=0.9,Pr=0.9,Ec=0.2,Sc=0.8,Ei=0.1,K=0.1,Bi=0.05,\beta^{*}=0.1$.

**Temperature of fluid particles and thermal memory effects:** The parameter γ is called the thermal relaxation parameter and has an analogy with the Deborah number proposed for viscoelastic fluid. This parameter determines the restoring characteristics of thermal equilibrium. It is important to mention here for γ=0, the non-Fourier’s law reduces to the classical Fourier’s law, and an increase of γ replicates the increase in ability of fluid to restore thermal equilibrium. Therefore, the temperature of fluid decreases (see Figure 10).

**Figure 11:**
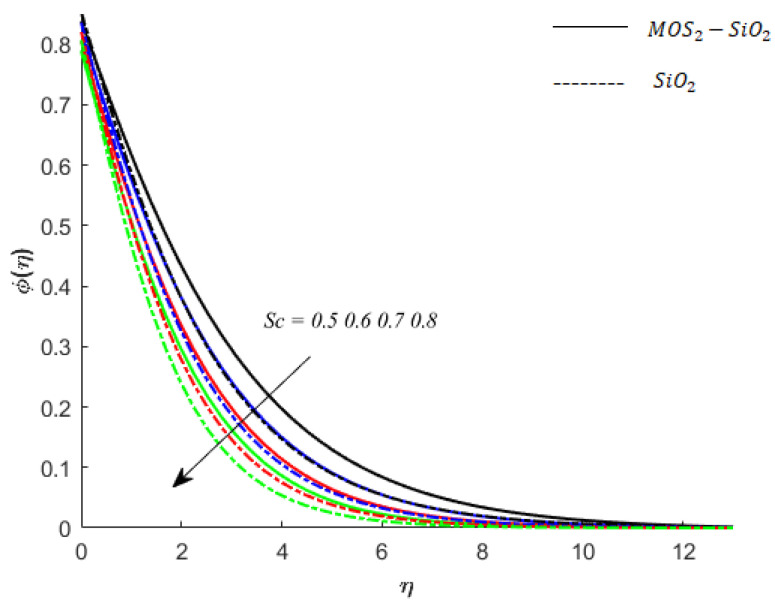
Influence of Sc on concentration when $M=0.9,Pr=0.9,Ec=0.2,K=0.1,Ei=0.1,Bi=0.05,\beta^{*}=0.1,\gamma =0.1,\gamma_{1}=0.1$.

**Concentration of fluid particles and Schmid number (**Sc**):** The Sc is the number that determines the impact of diffusion coeffect on the diffusion solute in the fluids. Here, in this work, the behavior of Sc on the diffusion of solute in $MoS_{2}-SiO_{2}-Casson\;fluid$ and $SiO_{2}-Casson\;fluid$ is examined, and the outcomes are presented by Figure 11.

**Figure 12:**
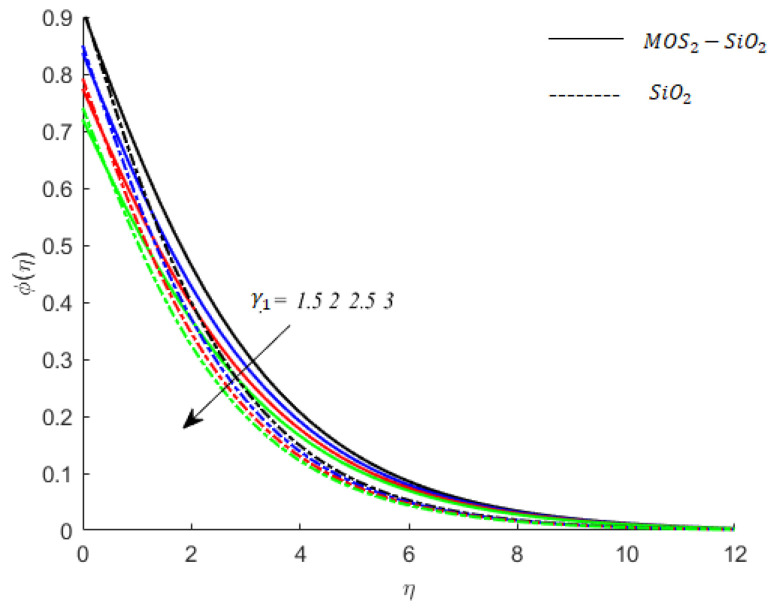
Influence of $\gamma_{1}$ on concentration when $M=0.9,Pr=0.9,Ec=0.2,Sc=0.8,K=0.1,Ei=0.1,Bi=0.05,\beta^{*}=0.1,\gamma =0.1$.

**Concentration of fluid particles and solutal relaxation time:** The effects of thermal relaxation time on the temperature of fluid particles are similar to the impact of solutal relaxation time on the concentration field (compare Figure 10 and Figure 12).

**Behaviors of wall shear rate of heat transfer and mass flux against selected parameters:** The behaviors of selected parameters $Bi,\;Pr,\;{\left(Gr\right)}_{t},\;\gamma \;\mathrm{and}\;\gamma_{1}$ on wall shear rate, heat-transfer rate and mass-transfer rate are examined. The results so obtained are recorded in the tabular numerical data (see Table 4) for both $SiO_{2}-Casson\;fluid$ (nanofluid) and $MoS_{2}-SiO_{2}-Casson\;fluid$. This table clearly depicts that heat transfer is enhanced due to simultaneous dissipation of nanostructures $MoS_{2}\;\mathrm{and}\;SiO_{2}$. Biot number Bi compels the wall shear stress to decrease in both types of fluids (nano and hybrid nanofluids). It is noticed that wall shear stress for the case of hybrid nanofluid ($MoS_{2}-SiO_{2}-Casson\;fluid$) is greater than that for the case of nanofluid ($SiO_{2}-Casson\;fluid$). An increasing rate of Bi on wall heat-transfer rate is observed. However, it has a decreasing trend for the case of mass transfer rate for both $SiO_{2}-\mathrm{Casson}$ and $MoS_{2}-SiO_{2}-Casson\;fluids$. Pr has shown an increasing behavior on wall shear stress and wall heat-transfer rate. However, the opposite trend is noticed for mass flux. For positive values of Grashof number ${\left(Gr\right)}_{t}$, the wall shear stress decreases; however, opposite trend is noticed for ${\left(Gr\right)}_{t}<0$. It is also found that for the assisting flow (${\left(Gr\right)}_{t}>0$), the rate of heat transfer and rate of mass transfer increase. The reverse behavior is noted for ${\left(Gr\right)}_{t}<0$. The trend of shear stress, mass flux and the rate of heat transfer for various values of $\gamma \;\mathrm{and}\;\gamma_{1}$ can be observed in Table 4.

## 5. Conclusions

The formulated models are solved numerically with the implementation of FEM. The Cattaneo–Christov generalized laws for heat and mass diffusion are helpful for analyzing thermal and solutal relaxation phenomena. The numerical samples were obtained for various values of related parameters to visualize the dynamics of flow quantities, including the rate of heat and mass transport flux. The main observations related to this study are

1. FEM provides convergent solutions.
2. The relaxation time phenomenon is responsible for the restoration of thermal and solutal changes, due to which, the thermal and solutal equilibrium states can be restored.
3. The phenomenon of yield stress is responsible for controlling momentum boundary layer thickness.
4. Porous medium exerts retarding force on the flow, and therefore, the deceleration inflow is observed.
5. The thermal efficiency of $MoS_{2}-SiO_{2}-$ *Casson fluid* is greater than the thermal efficiency of $SiO_{2}-$ *Casson fluid*.
6. The change in magnetic flux by the motion of the fluid particles results in a Lorentz force that opposes the flow, and the boundary layer thickness becomes shorter. It can be noticed also noticed that the Lorentz force associated with the motion of $SiO_{2}$—nanofluid has a smaller magnitude relative to the Lorentz force induced by the motion of hybrid nanofluid ($MoS_{2}\;–\;SiO_{2}$—nanofluid). Thus, the viscous region for momentum boundary layer flow nanofluid is wider than that for the flow of hybrid nanofluid.
7. The vortex viscosity is responsible for decreasing the macromotion of fluid particles. Thus, velocity of Newtonian fluid is greater than the velocity of micropolar fluid. Further, the impact of vortex motion of solid structures in the fluid on velocity of nanofluid is negligible in comparison with the case of hybrid nanofluid.
8. Joule heating associated with hybrid nanofluid flow has greater value in comparison with Joule heating in nanofluid. Thus, the temperature of hybrid nanofluid increases more rapidly than the temperature of nanofluid. Moreover, the Ohmic heating process in hybrid nanofluid is stronger than that in nanofluid.
9. Viscous dissipation results an increase in temperature of the fluids (both mono- and hybrid nanofluids).
10. The thermal relaxation time is responsible for decreasing the temperature of fluid. This is because of the ability of the fluid to restore thermal changes in order to attain thermal equilibrium.

**Future aspects:** In this study, $MoS_{2}$ and $SiO_{2}$ are used to enhance the thermal conductivity of the Casson fluid, and the optimized heat transfer is noticed. Based on the simulation, it is claimed that thermal performance is significantly increased. This observation is related to Casson fluid with the nanoparticles. However, it is very important to mention that this analysis should be performed for other non-Newtonian fluids. A similar analysis is also required for other nanoparticles. The comprehensive comparison between present study and the studies for other nanoparticles in other non-Newtonian fluids will give a comprehensive picture. Authors have the intension to do so in the future.

## Figures and Tables

**Table 1:** Values of properties of pure fluid and nanostructure.

Physical Property	Blood/Base Fluid	$MOS_{2}$	$SiO_{2}$
$\phi_{f}\left(nm\right)$	0.0	0.05	0.25
$\rho \left(Kg\backslash m^{3}\right)$	1060	5060	2200
k(W/(m.K))	0.429	34.5	1.2
$c_{p}\left(J/\left(kg.K\right)\right)$	3770	397.746	703
σ/(Ω/m)	$4.3\times 10^{-5}$	$2.6\times 10^{6}$	$10^{-27}$

**Table 2:** Local skin friction coefficient, Nusselt number and Sherwood number for hybrid nanofluid ($MOS_{2}-SiO_{2}$ ) and nanofluid ($SiO_{2}$ ) when $M=0.9,\;Pr=0.9,\;Sc=0.8,\;{\left(Gr\right)}_{c}=0.1,\;{\left(Gr\right)}_{t}=0.2,\;K=0.1,\;\alpha =0.3,\;\beta^{*}=0.1,\gamma =0.1,\gamma_{1}=0.1.$

e	$Re^{\frac{1}{2}}C_{f}$	$Re^{-\frac{1}{2}}Nu$	$Re^{-\frac{1}{2}}Sh$
10	2.6528312	0.3647946	0.3346214
50	2.9699726	0.3553285	0.3265893
100	2.9864624	0.3547900	0.3261574
150	2.9897546	0.3546829	0.3260747
200	2.9909392	0.3546445	0.3260456
250	2.9914955	0.3546265	0.3260320
300	2.9918005	0.3546166	0.3260247
350	2.9919856	0.3546106	0.3260202
400	2.9921062	0.3546067	0.3260173
450	2.9921892	0.3546040	0.3260153
500	2.9922388	0.3546021	0.3260139
550	2.9922630	0.3546007	0.3260129
600	2.9922866	0.3545001	0.3260121

**Table 3:** Comparison of temperature with Aninasaun et al. [32] for different values of Prandtl number when $\varphi_{1}=\varphi_{2}=0,\;{\left(Gr\right)}_{c}={\left(Gr\right)}_{t}=K=\gamma =\gamma_{1}=0$.

Pr	Aninasaun et al. [35]	Present Work
1	0.67650698	0.6751263
2	1.07352135	1.0755124
3	1.38075427	1.3808321

**Table 4:** Local skin friction coefficient, Nusselt number, and Sherwood number for hybrid nanofluid ($MOS_{2}-SiO_{2}$ ) and nanofluid ($SiO_{2}$ ) when $M=0.9,\;Sc=0.8,\;{\left(Gr\right)}_{c}=0.1,\;K=0.1,\;\alpha =0.3,\;Ei=0.1,\;\beta^{*}=0.1$.

		$\mathrm{Hybrid}\;\mathrm{Nanofluid}\;(MOS_{2}-SiO_{2})$			$\mathrm{Nanofluid}\;(SiO_{2})$		
		$Re^{\frac{1}{2}}C_{f}$	$Re^{-\frac{1}{2}}Nu$	$Re^{-\frac{1}{2}}Sh$	$Re^{\frac{1}{2}}C_{f}$	$Re^{-\frac{1}{2}}Nu$	$Re^{-\frac{1}{2}}Sh$
	0.3	2.983196	0.332254	0.327839	1.550508	0.331809	0.274995
Bi	0.4	2.975124	0.346307	0.327080	1.547106	0.343833	0.273122
	0.6	2.966398	0.361610	0.326245	1.543475	0.356726	0.271111
	0.9	2.960175	0.372594	0.325640	1.540915	0.365853	0.269686
	0.9	2.969972	0.355328	0.326589	1.544956	0.351459	0.271933
Pr	1.2	2.982838	0.407449	0.318419	1.556270	0.426194	0.254630
	1.5	2.995592	0.462848	0.310154	1.565150	0.492608	0.239609
	1.8	3.007677	0.518502	0.302151	1.572221	0.551755	0.226381
	0.2	2.931015	0.008746	0.371463	1.532676	0.171288	0.313080
${\left(Gr\right)}_{t}$	0.4	2.659066	0.058366	0.376682	1.409872	0.199231	0.315051
	0.6	2.401254	0.094303	0.381388	1.292906	0.220905	0.317027
	0.8	2.152702	0.121027	0.385802	1.180107	0.238387	0.318995
	0.8	3.091709	0.228875	0.401115	1.597960	0.235893	0.352285
γ	0.7	3.082004	0.215412	0.404166	1.594813	0.226250	0.355104
	0.6	3.071976	0.201547	0.407300	1.591568	0.216313	0.357992
	0.5	3.061600	0.187249	0.410520	1.588223	0.206071	0.360951
	3.0	2.994940	0.086758	0.511548	1.568195	0.132043	0.467676
$\gamma_{1}$	2.5	2.992579	0.088335	0.486832	1.566804	0.134235	0.442385
	2.0	2.990092	0.089963	0.461265	1.565340	0.136479	0.416249
	1.5	2.987473	0.091642	0.434826	1.563799	0.138768	0.389274

## Nomenclature

Symbols	Abbreviation
a	The stretching rate of sheet
	Cauchy stress tensor
P	Pressure
	Identity tensor
β	Casson fluid
μ	Dynamic viscosity
$A_{1}$	First Rivlin–Eriksen tensor
u, v	Velocity component
T	Temperature
C	Concentration
g	Gravitational acceleration
ρ	Density
$c_{p}$	Specific heat
k	Thermal conductivity
hnf	Hybrid nanofluid
D	Mass diffusion coefficient
$\tau_{o}$	Thermal relaxation time
$\tau_{1}$	Concentration relaxation time
σ	Electrical conductivity
$T_{\infty }$	Temperature at ambient position
$C_{\infty }$	Concentration at ambient position
M	Magnetic parameter
${\left(Gr\right)}_{t}$	Grashof number for temperature
${\left(Gr\right)}_{c}$	Grashof number for concentration
$\beta^{*}$	Heat generation parameter
K	Porous medium parameter
Pr	Prandtl number
Sc	Schmidt number
Ec	Eckert number
γ	Dimensionless thermal relaxation time parameter
$\gamma_{1}$	Dimensionless concentration relaxation time parameter
$\beta_{i}\;\mathrm{and}\;E_{i}$	Biot number for heat and mass, respectively
$C_{f}$	Skin friction
Nu	Nusselt number
Sh	Sherwood number
Re	Local Reynolds number
$w_{i}$	Weight function
φ	Volume fraction
FEM	Finite element method

## References

[1] Hamid M., Usman M., Khan Z., Ahmad R., Wang W. (2019). Dual solutions and stability analysis of flow and heat transfer of Casson fluid over a stretching sheet. Phys. Lett. A.

[2] Nadeem S., Haq R.U., Akbar N.S., Khan Z. (2013). MHD three-dimensional Casson fluid flow past a porous linearly stretching sheet. Alex. Eng. J..

[3] Mukhopadhyay S. (2013). Casson fluid flow and heat transfer over a nonlinearly stretching surface. Chin. Phys. B.

[4] Hayat T., Shehzad S.A., Alsaedi A. (2012). Soret and Dufour effects on magnetohydrodynamic (MHD) flow of Casson fluid. Appl. Math. Mech..

[5] Khan M.I., Waqas M., Hayat T., Alsaedi A. (2017). A comparative study of Casson fluid with homogeneous-heterogeneous reactions. J. Colloid Interface Sci..

[6] Sheikholeslami M., Rashidi M.M., Ganji D. (2015). Effect of non-uniform magnetic field on forced convection heat transfer of Fe3O4-water nanofluid. Comput. Methods Appl. Mech. Eng..

[7] Sheikholeslami M., Seyednezhad M. (2017). Nanofluid heat transfer in a permeable enclosure in presence of variable magnetic field by means of CVFEM. Int. J. Heat Mass Transf..

[8] Dogonchi A.S., Ganji D.D. (2016). Thermal radiation effect on the nano-fluid buoyancy flow and heat transfer over a stretching sheet considering Brownian motion. J. Mol. Liq..

[9] Dogonchi A.S., Chamkha A.J., Hashemi-Tilehnoee M., Seyyedi S.M., Haq R.-U., Ganji D.D. (2019). Effects of homogeneous-heterogeneous reactions and thermal radiation on magneto-hydrodynamic Cu-water nanofluid flow over an expanding flat plate with non-uniform heat source. J. Cent. South Univ..

[10] Ayub M., Malik M.Y., Ijaz M., AlQarni M.S., Alqahtani A.S. (2019). Cattaneo–Christov double-diffusion model for viscoelastic nanofluid with activation energy and nonlinear thermal radiation. Multidiscip. Model. Mater. Struct..

[11] Rana S., Nawaz M. (2019). Investigation of enhancement of heat transfer in Sutterby nanofluid using Koo–Kleinstreuer and Li (KKL) correlations and Cattaneo–Christov heat flux model. Phys. Scr..

[12] Ahmed S., Xu H. (2021). Forced convection with unsteady pulsating flow of a hybrid nanofluid in a microchannel in the presence of EDL, magnetic and thermal radiation effects. Int. Commun. Heat Mass Transf..

[13] Iftikhar N., Rehman A., Sadaf H. (2021). Theoretical investigation for convective heat transfer on Cu/water nanofluid and (SiO2-copper)/water hybrid nanofluid with MHD and nanoparticle shape effects comprising relaxation and contraction phenomenon. Int. Commun. Heat Mass Transf..

[14] Benkhedda M., Boufendi T., Tayebi T., Chamkha A.J. (2019). Convective heat transfer performance of hybrid nanofluid in a horizontal pipe considering nanoparticles shapes effect. J. Therm. Anal. Calorim..

[15] Aziz A., Jamshed W., Aziz T., Bahaidarah H.M.S., Rehman K.U. (2021). Entropy analysis of Powell–Eyring hybrid nanofluid including effect of linear thermal radiation and viscous dissipation. J. Therm. Anal. Calorim..

[16] Subhani M., Nadeem S. (2019). Numerical investigation into unsteady magnetohydrodynamics flow of micropolar hybrid nanofluid in porous medium. Phys. Scr..

[17] Waini I., Ishak A., Pop I. (2019). Flow and heat transfer along a permeable stretching/shrinking curved surface in a hybrid nanofluid. Phys. Scr..

[18] Kaneez H., Nawaz M., Alaoui M.K., Abdelmalek Z. (2020). An enhancement in transportation of heat energy in yield stress dusty fluid via mono and hybrid nanoparticles. Phys. Scr..

[19] Nawaz M., Rana S., Qureshi I.H., Hayat T. (2018). Three-dimensional heat transfer in the mixture of nanoparticles and micropolar MHD plasma with Hall and ion slip effects. AIP Adv..

[20] Nawaz M., Hayat T., Alsaedi A. (2012). Dufour and Soret effects on MHD flow of viscous fluid between radially stretching sheets in porous medium. Appl. Math. Mech..

[21] Arif U., Nawaz M., Alharbi S.O., Saleem S. (2021). Investigation on the impact of thermal performance of fluid due to hybrid nano-structures. J. Therm. Anal. Calorim..

[22] Sheikholeslami M., Ellahi R. (2015). Three dimensional mesoscopic simulation of magnetic field effect on natural convection of nanofluid. Int. J. Heat Mass Transf..

[23] Sheikholeslami M. (2017). Influence of Lorentz forces on nanofluid flow in a porous cylinder considering Darcy model. J. Mol. Liq..

[24] Rehman K.U., Saba N.U., Zehra I., Malik M.Y., Bilal S.M. (2020). Numerical study of thermal radiations and thermal stratification mechanisms in magnetohydrodynamic casson fluid-flow. Therm. Sci..

[25] Awais M., Awan S.E., Raja M.A.Z., Parveen N., Khan W.U., Malik M.Y., He Y. (2021). Effects of variable transport properties on heat and mass transfer in MHD bioconvective nanofluid rheology with gyrotactic microorganisms: Numerical approach. Coatings.

[26] Pal D. (2008). Heat and mass transfer in stagnation-point flow towards a stretching surface in the presence of buoyancy force and thermal radiation. Meccanica.

[27] Liu X., Xu X., Liu C., Ye J., Li H., Bai W., Dang C. (2017). Numerical study of the effect of buoyancy force and centrifugal force on heat transfer characteristics of supercritical CO_2_ in helically coiled tube at various inclination angles. Appl. Therm. Eng..

[28] Sheikholeslami M., Gorji-Bandpy M., Ganji D.D.G.-D., Rana P., Soleimani S. (2014). Magnetohydrodynamic free convection of Al2O3–water nanofluid considering Thermophoresis and Brownian motion effects. Comput. Fluids.

[29] Kandasamy R., Muhaimin I., Mohamad R. (2013). Thermophoresis and Brownian motion effects on MHD boundary-layer flow of a nanofluid in the presence of thermal stratification due to solar radiation. Int. J. Mech. Sci..

[30] Makinde O., Animasaun I. (2016). Thermophoresis and Brownian motion effects on MHD bioconvection of nanofluid with nonlinear thermal radiation and quartic chemical reaction past an upper horizontal surface of a paraboloid of revolution. J. Mol. Liq..

[31] Lin Y., Jiang Y. (2018). Effects of Brownian motion and thermophoresis on nanofluids in a rotating circular groove: A numerical simulation. Int. J. Heat Mass Transf..

[32] Animasaun I., Adebile E., Fagbade A. (2016). Casson fluid flow with variable thermo-physical property along exponentially stretching sheet with suction and exponentially decaying internal heat generation using the homotopy analysis method. J. Niger. Math. Soc..

[33] Alruwashid F.S., Dar M.A., Alharthi N.H., Abdo H.S. (2021). Effect of graphene concentration on the electrochemical properties of cobalt ferrite nanocomposite materials. Nanomaterials.

[34] Pal N., Sunwoo Y., Park J.-S., Kim T., Cho E.-B. (2021). Newly designed mesoporous silica and organosilica nanostructures based on pentablock copolymer templates in weakly acidic media. Nanomaterials.

[35] Nasr O., Jiang J.-R., Chuang W.-S., Lee S.-W., Chen C.-Y. (2021). Ag nanoparticle-decorated Cu_2_S nanosheets for surface enhanced raman spectroscopy detection and photocatalytic applications. Nanomaterials.

[36] Zemtsova E.G., Yurchuk D.V., Morozov P.E., Korusenko P.M., Kudymov V.K., Smirnov V.M. (2021). Features of the synthesis of the dispersed TiC phase with nickel nanostructures on the surface to create an aluminum-based metal composite. Nanomaterilas.

